# Comprehensive cross‐platform comparison of methods for non‐invasive EGFR mutation testing: results of the RING observational trial

**DOI:** 10.1002/1878-0261.12832

**Published:** 2020-11-13

**Authors:** Atocha Romero, Eloisa Jantus‐Lewintre, Beatriz García‐Peláez, Ana Royuela, Amelia Insa, Patricia Cruz, Ana Collazo, Javier Pérez Altozano, Oscar Juan Vidal, Pilar Diz, Manuel Cobo, Berta Hernández, Sergio Vázquez Estevez, Gretel Benítez, Maria Guirado, Margarita Majem, Reyes Bernabé, Ana Laura Ortega, Ana Blasco, Joaquim Bosch‐Barrera, Jose M. Jurado, Jorge García González, Santiago Viteri, Carlos Garcia Giron, Bartomeu Massutí, Ana Lopez Martín, Alejandro Rodriguez‐Festa, Silvia Calabuig‐Fariñas, Miguel Ángel Molina‐Vila, Mariano Provencio

**Affiliations:** ^1^ Liquid Biopsy Laboratory Biomedical Sciences Research Institute Puerta de Hierro‐Majadahonda Madrid Spain; ^2^ Medical Oncology Department Hospital Universitario Puerta de Hierro‐Majadahonda Madrid Spain; ^3^ CIBERONC Madrid Spain; ^4^ Mixed Unit TRIAL Príncipe Felipe Research Center & General University Hospital of Valencia Research Foundation Spain; ^5^ Biotechnology Department Universitat Politècnica de València Spain; ^6^ Laboratory of Oncology/Pangaea Oncology Quirón‐Dexeus University Hospital Barcelona Spain; ^7^ Biostatistics Unit CIBERESP Hospital Universitario Puerta de Hierro‐Majadahonda Madrid Spain; ^8^ Hospital Clínico Universitario de Valencia Spain; ^9^ Hospital la Paz Madrid Spain; ^10^ Hospital Universitario Sanchinarro Madrid Spain; ^11^ Hospital Virgen de los Lirios Valencia Spain; ^12^ Hospital Universitario La Fe Valencia Spain; ^13^ Complejo Asistencial Universitario de León Spain; ^14^ Hospital Regional Universitario Málaga Spain; ^15^ Complejo Hospitalario de Navarra Spain; ^16^ Hospital Universitario Lucus Augusti Lugo Spain; ^17^ Complejo Hospitalario Universitario Insular de Gran Canaria Las Palmas Spain; ^18^ Hospital General Universitario de Elche Alicante Spain; ^19^ Hospital de la Santa Creu i Sant Pau Barcelona Spain; ^20^ Hospital Virgen del Rocío Sevilla Spain; ^21^ Complejo Hospitalario de Jaén Spain; ^22^ Hospital Dr. Josep Trueta‐ICO Girona Spain; ^23^ Hospital Universitario Clínico San Cecilio Granada Spain; ^24^ Hospital Clínico Universitario de Santiago A Coruña Spain; ^25^ Instituto Oncológico Dr. Rosell Hospital Universitario Dexeus, Grupo Quiron Salud Barcelona Spain; ^26^ Hospital Universitario de Burgos Spain; ^27^ Hospital General Universitario Alicante Spain; ^28^ Hospital Severo Ochoa Leganés, Madrid Spain; ^29^ Department of Pathology Universitat de València Spain

**Keywords:** circulating free DNA, epidermal growth factor receptor, NGS, non‐small‐cell lung cancer, osimertinib, tyrosine kinase inhibitor

## Abstract

Several platforms for noninvasive *EGFR* testing are currently used in the clinical setting with sensitivities ranging from 30% to 100%. Prospective studies evaluating agreement and sources for discordant results remain lacking. Herein, seven methodologies including two next‐generation sequencing (NGS)‐based methods, three high‐sensitivity PCR‐based platforms, and two FDA‐approved methods were compared using 72 plasma samples, from *EGFR*‐mutant non‐small‐cell lung cancer (NSCLC) patients progressing on a first‐line tyrosine kinase inhibitor (TKI). NGS platforms as well as high‐sensitivity PCR‐based methodologies showed excellent agreement for EGFR‐sensitizing mutations (*K* = 0.80–0.89) and substantial agreement for T790M testing (*K* = 0.77 and 0.68, respectively). Mutant allele frequencies (MAFs) obtained by different quantitative methods showed an excellent reproducibility (intraclass correlation coefficients 0.86–0.98). Among other technical factors, discordant calls mostly occurred at mutant allele frequencies (MAFs) ≤ 0.5%. Agreement significantly improved when discarding samples with MAF ≤ 0.5%. *EGFR* mutations were detected at significantly lower MAFs in patients with brain metastases, suggesting that these patients risk for a false‐positive result. Our results support the use of liquid biopsies for noninvasive *EGFR* testing and highlight the need to systematically report MAFs.

## Graphical abstract

Plasma samples from 72 *EGFR*‐mutant advanced NSCLC patients, collected upon progression to first‐line TKI, were analyzed by seven methodologies (two NGS‐based methods, three high‐sensitivity PCR‐based platforms, and two FDA‐approved methods). Our study demonstrates a good to excellent agreement between methodologies and supports the use of liquid biopsies for therapy decision‐making.

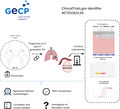

**Abbreviations**
BEAMing: beads, emulsion, amplification, and magnetics
cfDNA: circulating free DNA, cell‐free DNA
cobas: cobas^®^ *EGFR* Mutation Test v2 (Roche Diagnostics)
ctDNA: circulating tumor DNA
CUSUM: cumulative sum
ddPCR: droplet digital polymerase chain reaction
dPCR: digital polymerase chain reaction
EGFR: epidermal growth factor receptor
FFPE: formalin‐fixed, paraffin‐embedded
ICC: intraclass correlation coefficient
MAF: mutant allele frequency
NGS platforms: Ion S5™ XL and GeneRead™
NGS: next‐generation sequencing
NSCLC: non‐small‐cell lung cancer
PNA‐Q‐PCR: peptic nucleic acid probe‐based real‐time polymerase chain reaction
Therascreen: Therascreen *EGFR* Plasma RGQ PCR Kit (QIAgen)
TKI: tyrosine kinase inhibitor

## 1 Introduction

Tyrosine kinase inhibitors (TKIs) have dramatically changed the outcome of patients with *EGFR*‐positive non‐small‐cell lung cancer (NSCLC) [1, 2, 3, 4]. Osimertinib, a third‐generation TKI, is currently the standard of care as second‐line treatment in patients with T790M‐positive tumors at progression to first‐ or second‐generation EGFR TKI [5] as well as for first‐line treatment of EGFR‐positive NSCLC patients [6]. Biomarker testing using formalin‐fixed, paraffin‐embedded (FFPE) samples remains the reference standard, yet this approach may be limited by the availability of tumor and quality of DNA. Conversely, there is considerable evidence demonstrating that cell‐free DNA (cfDNA) genotyping represents a viable and faster approach [7, 8]. In this way, cfDNA testing is recommended for guiding first‐ and second‐line treatment in specific clinical circumstances, most notably, when a patient is medically unfit for invasive tissue sampling or when following pathologic confirmation of a NSCLC diagnosis, there is no sufficient material for molecular testing. Indeed, guidelines recommend testing the T790M resistance mutation in the blood after progression to an *EGFR* TKI as a first choice, and rebiopsies are suggested in case of a negative result [9]. As a result, *EGFR* genotyping using plasma samples is becoming widely used in the clinical setting. However, *EGFR* mutation detection in plasma samples is subject to the sensitivity of the method used, which may limit the access to targeted therapies.

Currently, several platforms are available for noninvasive *EGFR* testing in blood, some of which have received approval from the U.S. Food and Drug Administration (FDA) as well as European Medicines Agency (EMA) as companion kits for *EGFR* TKIs. Notably, the reported sensitivities of the different assays for *EGFR* mutation detection in the cfDNA from patients with advanced NSCLC vary as much as from 30% to 100% [10]. While there are numerous reports on the sensitivity and specificity of different platforms using tissue as a gold standard [11, 12], studies evaluating the agreement between different methods for liquid biopsy analysis in large prospective cohorts remain limited, and reports comparing quantitative measurements of mutant allele frequencies (MAFs) are particularly scarce.

In this study, we describe the results of an observational trial specifically designed to evaluate the agreement between seven methods for the detection of *EGFR* mutations in blood, including two next‐generation sequencing (NGS)‐based methods, three high‐sensitivity PCR‐based platforms, and two FDA‐approved methods. To our knowledge, this is the largest study (in terms of the number of platforms evaluated) so far to formally compare available technologies designed for noninvasive *EGFR* mutation testing and the first to comprehensively evaluate the concordance between MAFs. In addition, causes for discordant results are thoroughly investigated.

## 2 Methods

### 2.1 Study cohort

This is a non‐post‐authorization (non‐PAS) and noninterventional prospective, multicentre, cross‐sectional study (ClinicalTrials.gov Identifier: NCT03363139) in which 72 patients with *EGFR*‐mutant NSCLC were enrolled between July 2018 and January 2019 in 23 Spanish hospitals. There was no intention for validation of any technique in the study. The study protocol was approved by the Hospital Puerta de Hierro Ethics Committee (internal code PI‐154/17), and informed consent was obtained from all participants. Study monitoring was carried out by the Spanish Lung Cancer Group (www.gecp.org). Inclusion criteria were as follows: (a) patients diagnosed with EGFR‐mutant, stage IIIB and IV non‐small‐cell lung cancer, who have progressed as assessed by CT scan according to RECIST criteria v.1.1 to first‐ or second‐generation EGFR tyrosine kinase inhibitors (TKIs) (e.g., gefitinib, erlotinib, afatinib), including patients who received a chemotherapy line before TKI treatment. Samples must be drawn before the patient starts a new treatment. (b) Patients must sign the informed consent of the study. (c) Patients aged ≥ 18 years. Exclusion criteria were as follows: (a) patients progressing to third‐generation EGFR TKIs (e.g., osimertinib) and (b) no possibility of venipuncture. The patients participating in this noninterventional study did not receive treatment in relation to the study. Prospective information about treatments was not collected.

Three blood samples blood samples were collected per patient upon disease progression after a first‐line treatment with a first‐ or second‐generation TKI, assessed by RECIST criteria v. 1.1, and before the initiation of second‐line treatment. Median time between the assessment of progressive disease and blood extraction was below 4 weeks in all cases. Eligible patients were both male and female, age > 18 years, with a pathologically confirmed diagnosis of stage IV NSCLC harboring an *EGFR* mutation, and who had progressed with first‐line *EGFR* TKI treatment. In all cases, whole‐blood samples were collected in a 10‐mL Streck Cell‐Free DNA BCT tube (Streck, La Vista, NE, USA) and in two 8.5‐mL PPT™ tubes (Becton Dickinson, Franklin Lakes, NJ, USA). Samples were first sent to a central laboratory 1 (L1) for storage, processing, and distribution of the plasma samples to the other two central laboratories (L2 and L3). In all cases, the oncologist was blinded to laboratory result. L1, L2, and L3 were blinded for *EGFR* mutation status.

### 2.2 Laboratory procedures

Plasma was separated from the cellular fraction by two consecutive centrifugations at 1600 ***g*** for 10 min and at 6000 ***g*** for 10 min. Samples were then divided into 6 aliquots of 2.0 mL and stored at −80 °C, until further analysis or distribution to L2 and L3.

### 2.3 DNA extraction

In order to compare cfDNA yield between different extraction methods, all samples were processed with the following methods: (a) Maxwell^®^ RSC (MR) ccfDNA Plasma Kit (Promega Corporation, Madison, WI, USA), (b) QIAamp Circulating Nucleic Acid (QCNA) (QIAgen, Valencia, CA, USA), and (c) QIAsymphony DSP Virus/Pathogen Midi Kit using a QIAsymphony (QS) robot (QIAgen), in all cases following the manufacturer's instructions. The input volume as well as the final elution volume per method is presented in Table S1. cfDNA concentration was measured using the Qubit dsDNA HS Assay kit (Thermo Fisher Scientific, Waltham, MA, USA) on a Qubit 2.0 Fluorometer (Thermo Fisher Scientific).

### 2.4 cfDNA genotyping

The presence of *EGFR* mutations in the purified cfDNA was evaluated in the 72 samples using 7 different methods, namely cobas^®^ *EGFR* Mutation Test v2 (Roche Diagnostics, Penzberg, Germany), Therascreen *EGFR* Plasma RGQ PCR Kit (QIAgen), OncoBEAM *EGFR* (Sysmex Inostics, Hamburg, Germany), QuantStudio^®^ 3D Digital PCR System (Applied Biosystems, South San Francisco, CA, USA), a 5′‐nuclease real‐time PCR (TaqMan^®^, Thermo Fisher Scientific) assay in presence of a peptic nucleic acid probe (PNA‐Q‐PCR), and two NGS platforms (Ion S5™ XL and GeneRead™) using two different gene panels, Oncomine™ Pan‐Cancer Cell‐Free Assay (Thermo Fisher, Palo Alto, CA, USA) and QIAact Lung DNA UMI Panel (QIAgen). Figure S1 shows the study flowchart, indicating which methods were used in each of the three laboratories. The limit of detection (LOD) of each method in terms of MAF is presented in Table S2.

#### 2.4.1 FDA‐approved methods

Cobas^®^ EGFR Mutation Test v2 (Roche Diagnostics) and Therascreen EGFR Plasma RGQ PCR Kit (QIAgen) were used according to the instructions of the manufacturers.

#### 2.4.2 OncoBEAM™ EGFR kit (Sysmex^®^)

BEAMing is a highly sensitive and quantitative platform based on multiplex PCR (mPCR) targeting somatic alterations and followed by a second PCR amplification performed on magnetic beads compartmentalized in millions of oil emulsions). All experiments were performed according to the supplier's recommendations. Briefly, 125 μL of cfDNA samples were employed for mPCR. After mPCR, six replicates of each sample were mixed in another plate to perform nested‐PCR. Then, serial dilutions (1 : 50/1 : 60/1 : 50) were carried out. The diluted samples were then transferred to the emulsion PCR plate, together with emulsion working reagents (one for each codon). After this step, the EmulsiFIRE solution was added to induce the emulsion, creating millions of PCR compartments (hydrophilic droplets with a single magnetic bead inside). After breaking the emulsion PCR, WT and mutant‐specific probes were hybridized and analyzed by flow cytometry using the Cube 6i cytometer of Sysmex^®^ (Sysmex Inostics). Plasma was considered positive by BEAMing for a given mutation if the mutation was detected above thresholds used for clinical application (Table S2).

#### 2.4.3 QuantStudio^®^ 3D Digital PCR (dPCR)

Cell‐free DNA was analyzed using commercially available predesigned TaqMan^®^ Liquid Biopsy dPCR assays on a QuantStudio^®^ 3D Digital PCR System (Applied Biosystems). The dPCR was carried out on a final volume of 18 μL and using 8.55 μL of cfDNA template. Subsequently, 14.5 μL was loaded into a QuantStudio 3D Digital PCR 20K chip. The cycling conditions were as follows, initial denaturation at 96 °C for 10 min, followed by 40 cycles at 56 °C for 2 min, and at 98 °C for 30 s, a step of 72 °C for 10 min, and finally, samples were maintained at 22 °C for at least 30 min. Chip fluorescence was read twice. Results were analyzed with quantstudio^®^ 3d analysis suite™ cloud Software (Thermo Fisher Scientific). The automatic call assignments for each data cluster were manually adjusted when needed. The result of the assay is reported as the ratio of mutant DNA molecules relative to the sum of mutant and wild‐type (wt) DNA molecules. A negative control DNA was included in every run. Details about assay performance have been described elsewhere [13]. Mutations detected by this assay include p.L747_T751>P c.2239_2251>C; p.L747_A750>P c.2238_2248>GC; p.E746_T751delELREAT c.2236_2253del18; p.L747_T751delLREAT c.2238_2252del15; p.E746_A750delELREA c.2235_2249del15 c.2236_2250del15; p.L747‐S753>S c.2240_2257del18; p.L858R c.2573T>G 2573_2574TG>GT; p.G719A c.2156G>C; p.T790M c.2369C>T; p.G719S c.2155G>A; p.G719C c.2155G>T; p.L747_T751delLREAT c.2239_2253del15; p.L747_S752delLREATS c.2239_2256del18; p.L861Q c.2582T>A; p.C797S c.2390G>C; 2389T>A; p.E746‐S752>V c.2237_2255>T; p.L747_P753>Q c.2239_2258>CA.

#### 2.4.4 PNA‐Q‐PCR

The assay is based on quantitative real‐time PCR (TaqMan) in the presence of a PNA clamp (Eurogentec, Seraing, Belgium) designed to inhibit the amplification of the wild‐type alleles. The assay has been fully validated, has an ISO15189 accreditation, and allows estimation of the absolute and relative abundances of mutant alleles in positive samples. Briefly, amplification is performed in a final volume of 12.5 μL, using 3 μL (~ 4.5 ng) for exon 21 analysis or 1 μL (~ 1.5 ng) for exon 19 and p.T790M analysis of cfDNA 6.25 μL of Genotyping Master Mix (Applied Biosystems, Foster City, CA, USA) 0.96 pmol of each primer 1.2 pmol of probes and 6.25 pmol (for exon 21 and p.T790M) or 62.4 pmol (for exon 19) of PNA. Samples are submitted to 50 cycles of 15 s at 92 °C and 1.5 min at 60 °C, in a QuantStudio™ 6 real‐time PCR System (Applied Biosystems/Thermo Fisher Scientific). The sequence of the primers, probes, and PNAs used and analytical performance has been described elsewhere [10, 14]. Specifically, the assay covers the following mutations: p.L747‐T751 c.2238_2252del15; p.L747_S753>S c.2240_2257del18; p.E746_A750delELREA c.2235_2249del15, c.2236_2250del15; p.L747_S752delLREATS c.2239_2256del18; p.E746_S752>V c.2237_2255>T; p.E746_T751>A c.2237_2244>T; p.L747_T751>P c.2239_2251>C; p.L747_A750>P c.2239_2248TTAAGAGAAG>C; p.L858R c.2573T>G 2573_2574TG>GT; p.G719A c.2156G>C; p.T790M c.2369C>T; p.G719S c.2155G>A; p.G719C c.2155G>T; p.L861Q c.2582T>A; p.C797S c.2390G>C. Analyses were carried out in duplicate using one sample of purified cfDNA, when possible. In addition, all samples were assayed in the absence of PNA to confirm the presence of cfDNA. Genomic DNAs from cell lines at 1.5 ng·μL^−1^ were used as positive and negative controls. Extraction and nontemplate controls were added in each run. A sample was considered positive if the same mutant allele amplified in the two duplicates in the presence of PNA. If amplification was only detected in one duplicate, samples were reanalyzed and considered positive if again at least one of the duplicates was positive for the same mutated allele.

#### 2.4.5 NGS with the oncomine pan‐cancer cell‐free assay (Oncomine)

Library preparation was performed according to manufacturer's instructions. All the purifications were made using AMPure XP magnetic beads (Beckman Coulter, Brea, CA, USA). Library quantification was performed using Ion Library TaqMan^®^ Quantitation kit (Thermo Fisher) in a StepOnePlus™ qPCR machine (Thermo Fisher). The individual libraries were diluted to a final concentration of 100 pm. The final barcoded libraries were pooled and adjusted to a final concentration of 50 pm. Template preparation and chip loading were carried out on an Ion Chef™ System (Thermo Fisher). Eight samples were loaded onto an Ion 550™ chip. Finally, Ion 550™ chips were sequenced on an Ion S5™ Sequencer (Thermo Fisher). Analysis of raw sequencing data was performed using torrent suite Software (v5.10.0, Thermo Fisher). For sequencing coverage analysis, the coverageanalysis (v.5.10.0.3) plug‐in was used (Thermo Fisher). Raw reads were aligned to the human reference genome hg19. Variant calling, annotation, and filtering were performed on the Ion Reporter (v5.10) platform using the Oncomine TaqSeq Pan‐Cancer Liquid Biopsy workflow (v5.10). The clinical significance of somatic variants was performed according to Standards and Guidelines for the Interpretation and Reporting of Sequence Variants in Cancer.

#### 2.4.6 NGS with the GeneReader platform (GeneReader)

Purified DNA (16.75 μL, ~ 10–70 ng) was used as a template to generate libraries for sequencing with the GeneRead TM QIAact Lung DNA UMI Panel, according to manufacturer's instructions. The panel is designed to enrich specific target regions containing 550 variant positions in 19 selected genes frequently altered in lung cancer tumors (AKT1, ALK, BRAF, DDR2, EGFR, ERBB2/HER2, ESR1, KIT, KRAS, MAP2K1, MET, NRAS, NTRK1, PDGFRA, PIK3CA, PTEN, ROS1, FGFR1, and RICTOR), including MET exon 14 skipping mutations. Libraries were quantified using a QIAxcel Advanced System (QIAgen) and Qubit dsDNA HS Assay kit (Thermo Fisher Scientific), diluted to 100 pg/μL, and pooled in batches of 6 (liquid biopsies). Clonal amplification was performed on 625 pg of pooled libraries by the GeneRead Clonal Amp Q Kit (QIAgen) using the GeneRead QIAcube and an automated protocol. Following bead enrichment, pooled libraries were sequenced using the GeneRead UMI Advanced Sequencing Q kit in a GeneReader instrument. qiagen clinical insight analyze software (QIAgen) was employed to perform the secondary analysis of fastq reads, align the read data to the hg19 reference genome sequence, call sequence variants, and generate a report for visualization of the sequencing results. Variants were imported into the QIAGEN Clinical Insight Interpret web interface for data interpretation and generation of final custom report.

### 2.5 Statistical analysis

For the analysis of cfDNA extraction yield according to extraction method, a Friedman test was carried out to assess whether the measurements of the amount of cfDNA (ng) normalized by milliliter of plasma obtained by each methodology were equivalent. The Friedman test is used for one‐way repeated‐measures analysis of variance by ranks [15]. The difference in AFs between EGFR‐sensitizing and p.T790M mutation was assessed using the Wilcoxon signed‐rank test. The agreement between different methodologies for the assessment of p.T790M status as well as the original EGFR‐sensitizing mutation status (detected vs not detected) was evaluated using the kappa coefficient values and the corresponding 95% confidence intervals (95% CI). The strength of agreement is considered slight when the *K* values are between 0.00 and 0.20 fair, 0.21 and 0.40 moderate, 0.41 and 0.60 good, 0.61 and 0.80 and almost perfect, 0.81 and 0.99 [16]. To evaluate the relative accuracy of each method, EGFR mutation status using the FFPE sample from the rebiopsy was considered the gold standard. For this purpose, sensitivity, specificity, positive likelihood ratio, and negative likelihood ratio were calculated.

The difference in MAFs between *EGFR* mutations was assessed using the Wilcoxon signed‐rank test and the reliability between MAFs measured by different methodologies was evaluated using the intraclass correlation coefficient (ICC) through a two‐way mixed‐effects model, along with the 95% confidence interval. Lin's concordance correlation coefficient [17, 18] was estimated to assess the concordance between the measures two by two, interpreting the coefficient as poor if < 0.90, 0.90–0.95 as moderate, 0.95–0.99 as substantial and > 0.99 almost perfect [19].

In addition, Passing and Bablok [20] regression analysis was performed to assess the agreement and possible systematic bias between methods. Linear model validity was performed using the CUSUM test for linearity. Similarly, AF measurements by NGS‐based methodologies employed were graphically displayed by means of Bland–Altman plot [21].

Statistical significance was set at *P* < 0.05. The statistical analysis was performed using stata v.15.1 software (StataCorp. 2017; Stata Statistical Software: Release 15, College Station, TX, USA: StataCorp LLC.)

## 3 Results

### 3.1 Study cohort

Clinico‐pathological characteristics of the study population are presented in Table 1. Most patients were women (66.67%), of Caucasian ethnicity (95.83%), age range at diagnosis from 37 to 84 years, with an average of 65.5 years, and 58.33% of the patients were never smokers. According to the pathology reports, 91.67% of the cases were adenocarcinomas; the rest of the cases (8.33%) corresponded to adenosquamous and large‐cell carcinoma. All patients were stage IV, with 69% being stage IVA. Regarding EGFR status, 48.61% harbored a deletion in exon 19, 45.83% a point mutation in exon 21, 2.78% a point mutation in exon 18, one tumor harbored S768I in exon 20, and another tumor harbored an insertion in exon 20. There were no significant differences in mutation distribution according gender, age, or histology. Finally, the median number of metastatic lesions at disease progression was 3 (range 1–12), and 27.8% of patients had central nervous system (CNS) involvement.

**Table 1:** Clinicopathological characteristics of the study population.

Feature	Value	%
Age (years)	66	
Sex (*N*)		
Male	24	33
Female	48	67
Smoking status (*N*)		
Current/ex	30	42
Never	42	58
Histology (*N*)		
Adenocarcinoma	66	92
Other	6	8
Stage		
IVA	50	69
IVB	22	31
EGFR mutation (*N*)		
Deletion exon 19	35	49
Point mutation exon 21	33	46
Other	4	6
Treatment		
Afatinib	20	28
Erlotinib	19	26
Gefitinib	31	43
Other	2	3

### 3.2 Comparison of DNA extraction methods

Plasma samples were aliquoted and used for cfDNA extraction using three different methods: MR, QCNA, and QS. There was a moderate to strong correlation between the amount of cfDNA (ng·mL^−1^ plasma) obtained by each methodology (Pearson correlation coefficients: 0.85, 0.89, and 0.98 for QCNA‐MR, MR‐QS, and QCNA‐QS comparisons, respectively; *P* < 0.05 in all cases). The median total amounts of cfDNA (ng) normalized by plasma input volume, as well as the P25 and P75 yielded by each method, are presented in Table 2. There were significant differences in the cfDNA isolation yield between the methods evaluated (*P *< 0.001), being lower for MR method compared to QCNA and QS. Total amount of cfDNA obtained for each patient and according to each methodology is presented in Table S3.

**Table 2:** Extraction yield. Median, P25 and P75 of the amount (ng) of cfDNA obtained per mL of plasma.

	Extraction method		
	QCNA	MR	QS
Median	19.71	12.25	18.25
P25	13.91	6.78	13.47
P75	28.68	24.06	25.50

### 3.3 Agreement between methods for EGFR mutation detection

**Fig. 1:**
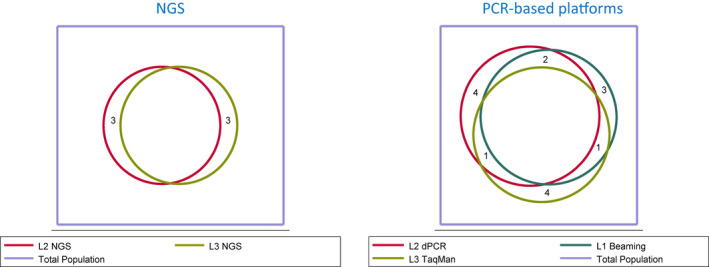
Venn diagrams showing concordance among NGS‐based methodologies and PCR‐based platforms for T790M detection. L2 NGS: Oncomine™ Pan‐Cancer Cell‐Free Assay performed in laboratory 2. L3 NGS: GeneRead™ QIAact Lung DNA UMI Cancer Panel performed in laboratory 3. L2 dPCR: QuantStudio^®^3D Digital PCR System, performed in laboratory 2. L3 TaqMan in‐house 5‐nuclease real‐time PCR assay in presence of PNA carried out in laboratory 3. L1 BEAMing OncoBEAM*EGFR*performed in laboratory 1.

Purified cfDNA samples were analyzed in three central laboratories using a total of seven methods. The proportion of observed agreement and Cohen's kappa index (K) between methods is shown in Table 3. The agreement between all methods was almost perfect for the detection of deletions in exon 19 (*K* = 0.87; 95% CI: 0.78–0.96) and substantial for exon 21 point mutations (*K* = 0.76; 95% CI: 0.63–0.89). Regarding the T790M resistance mutation, concordance was lower but still substantial (*K* = 0.68; 95% CI: 0.57–0.79). The comparison of two FDA‐approved methods (cobas^®^ and Therascreen) showed almost perfect agreement for the detection of exon 19 deletions and T790M mutation (*K* = 0.81; 95% CI: 0.65–0.97 and *K* = 0.83; 95% CI: 0.66–1.00, respectively) and substantial agreement for the identification of point mutations in exon 21 (*K* = 0.61; 95% CI: 0.40–0.82). Remarkably, the agreement between NGS platforms for the detection of *EGFR*‐activating mutations as well as the T790M mutation was particularly good (Fig. 1) (*K* = 0.84; 95% CI: 0.68–1.00, *K* = 0.83; 95% CI: 0.66–1.00 and *K* = 0.77; 95% CI: 0.59–0.95 for the detection of mutations in exon 19, 21, and T790M, respectively). Finally, high‐sensitivity PCR‐based methodologies showed perfect to substantial agreement (*K* = 0.89; 95% CI: 0.78–0.99, *K* = 0.80; 95% CI: 0.67–0.92 and *K* = 0.68; 95% CI: 0.54–0.83 for the detection of mutations in exon 19, 21, and T790M, respectively).

**Table 3:** Agreement between methodologies for the detection of deletions in exon 19, point mutations in exon 21 and the T790M mutation. Percentage of agreement, Cohen's kappa index (K), and corresponding confidence intervals.

Comparison group	Mutation	Agreement	Kappa	95% CI
All techniques	Exon 19	95.50	0.87	0.78–0.96
	Exon 21	91.26	0.76	0.63–0.89
	T790M	86.83	0.68	0.57–0.79
IVD‐approved	Exon 19	92.86	0.81	0.65–0.97
	Exon 21	85.71	0.61	0.40–0.82
	T790M	94.29	0.83	0.66–1.0
High‐sensitivity PCR based	Exon 19	96.15	0.89	0.78–0.99
	Exon 21	91.67	0.80	0.67–0.92
	T790M	85.19	0.68	0.54–0.83
NGS‐based	Exon 19	94.37	0.84	0.68–1.00
	Exon 21	94.2	0.83	0.66–1.00
	T790M	91.55	0.77	0.59–0.95

### 3.4 Investigation of discordant results

The possible causes of discordant results were investigated by elucidating the relationship between the detection of *EGFR* mutations and cfDNA input (ng) and time between assessment of progressive disease and blood drawn, but no significant differences in cfDNA input or withdrawn timing were found between concordant and discordant samples. Next, we examined whether discordant results were related to differences in the limits of detection (LOD) of the different assays and, consequently, if discordant calls occurred mostly at low MAFs. We thus performed an agreement analysis discarding samples in which *EGFR* mutations were detected at MAFs ≤ 0.5%. It is noteworthy that, in this subset of samples, the agreement between high‐sensitivity PCR‐based methods was perfect for the detection of exon 19 deletions and the T790M mutation and increased to *K* = 0.93 (95% CI: 0.83–1.00) in the case of point mutations in exon 21 (Table S4). The agreement for exon 21 and T790M mutations also improved in the case of NGS‐based methods when samples with MAFs ≤ 0.5% were excluded from the analysis.

Finally, we investigated whether any clinico‐pathological feature of the patients was associated with lower MAFs and could be a potential indicator of tumor shedding. In this study, ctDNA levels were not dependent on age, sex, histology, sum of metastatic lesions, or metastasis location, except for the presence of CNS metastasis. *EGFR‐*sensitizing mutations were detected at significantly lower MAFs in patients progressing exclusively at the CNS level compared to patients with disease progression assessed at other anatomical locations (Fig. S2). The T790M detection rate was also lower in the subset of patients progressing at CNS exclusively (Table S5).

### 3.5 Correlation between MAFs obtained by different methods

In our patient cohort, MAFs of positive samples ranged from 0.02% to 63.9%. A list containing all detected mutations and corresponding MAFs is available in Data S1. We assessed the reliability of MAFs obtained using quantitative techniques by first calculating the intraclass correlation coefficients (ICCs). As shown in Table 4, the MAF results determined by the two NGS‐based methods, Oncomine™ Pan‐Cancer Cell‐Free Assay and GeneRead™ QIAact Lung DNA UMI Cancer Panel, were almost identical (ICC = 0.98; 95% CI: 0.96–0.99 for *EGFR*‐sensitizing mutations and ICC = 0.97; 95% CI: 0.95–0.98 for T790M). Similarly, MAFs estimated using high‐sensitivity PCR‐based platforms showed an excellent agreement (ICC = 0.93; 95% CI: 0.90–0.96 for *EGFR*‐sensitizing mutations and ICC = 0.86; 95% CI: 0.79–0.91 for T790M).

**Table 4:** ICC and corresponding confidence intervals for equivalent MAF measurements.

Comparison group	Mutation	ICC	95% CI
All techniques	Sensitizing	0.94	0.92–0.96
	T790M	0.94	0.91–0.96
High‐sensitivity PCR based	Sensitizing	0.93	0.90–0.96
	T790M	0.86	0.79–0.91
NGS‐based	Sensitizing	0.98	0.96–0.99
	T790M	0.97	0.95–0.98

**Fig. 2:**
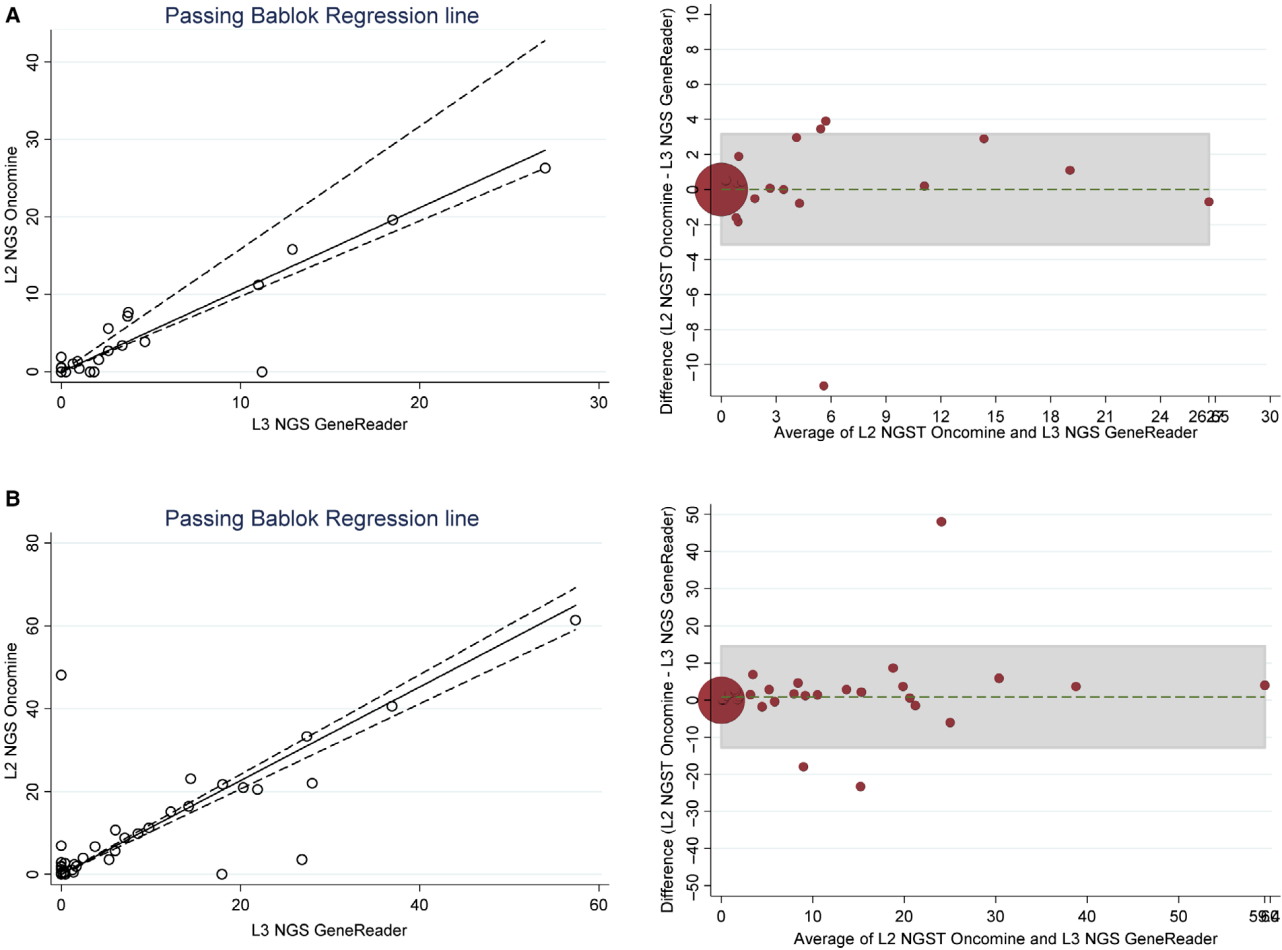
Comparison of MAFs obtained by NGS‐based platforms. (A) Passing‐Bablok regression showing close concordance between the two methods for the assessment of T790M MAFs and Bland–Altman plot showing low level of bias between both methods for quantifying T790M allele frequency. (B) Passing–Bablok regression and Bland–Altman plot showing the agreement between NGS‐based platforms for the quantification of*EGFR*‐sensitizing mutations.

Next, we used Passing–Bablok regression analysis to estimate the agreement and possible systematic bias between NGS‐based methods and obtained equations of *Y* = 1.107 (95% CI: 1.029–1.200) X for EGFR‐activating mutations and *Y* = 1.059 (95% CI: 0.974–1.585) X for MAFs of the T790M mutation (Fig. 2A). A CUSUM test indicated no significant deviation from linearity (*P* > 0.20), while the Bland–Altman plot (Fig. 2B) showed little bias between the two NGS‐based methods for EGFR‐sensitizing mutation and T790M MAF quantification (bias = 0.85, 95% CI = −12.99 to −14.69 and bias = 0.013, 95% CI = −3.12–3.14, respectively), with only three measurements lying outside the confidence interval in both cases. Similar results were obtained for PCR‐based platforms (Fig. S3).

Finally, there was a significant but weak positive correlation between MAFs and total cfDNA concentration (Spearman's correlation coefficient below 0.4 in all cases).

### 3.6 Comparison with tissue biopsies

A rebiopsy at disease progression was obtained from 35 (48.61%) patients. According to the pathology report, the original *EGFR‐*sensitizing mutation was detected in all FFPE samples, while the T790M mutation was present in 15 tumor samples (43%). The T790M mutation was more frequent in patients whose tumors harbored a deletion in exon 19 (77%) than in those with other sensitizing mutations (54%). Of note, in cases in which rebiopsy was not possible (*N* = 37), the T790M mutation was detected in the plasma sample by at least one method in 18 cases. Overall, T790M mutation was detected by at least one method in 39 (54%) plasma samples. The detection of the T790M mutation in the plasma was not associated with the TKI received nor with any clinic‐pathological features analyzed (gender, age, histology, smoking status).

The sensitivity, specificity, and positive and negative likelihood ratios for each method, considering the results of rebiopsy as the gold standard, are shown in Table 5. Of note, the G719X and S768l mutations, which were reported to be present in two rebiopsies from two independent patients, were not detected by any of the methodologies. As shown, all parameters were superior for *EGFR*‐sensitizing mutations compared with the T790M mutation. Remarkably, the T790M was systematically detected at lower MAF in the plasma sample than the original *EGFR*‐sensitizing mutation (*P* < 0.001; Wilcoxon test) (Fig. S4), suggesting that the lower MAF values for T790M could limit assay performance.

**Table 5:** Sensitivity, specificity positive predictive value (PPV) and negative predictive value (NPV) of each methodology and according to the type of mutation.

Methodology	T790M				Exon 21				Exon 19			
	Sensitivity (%)	Specificity(%)	PPV (%)	NNV(%)	Sensitivity (%)	Specificity(%)	PPV (%)	NNV(%)	Sensitivity (%)	Specificity(%)	PPV (%)	NNV(%)
BEAMing	54	73	81	42	80	100	100	92	71	100	100	70
dPCR	58	73	82	44	90	100	100	96	62	100	100	64
PNA‐Q‐PCR	54	73	81	42	70	100	100	89	53	100	100	58
NGS Oncomine	42	90	91	40	89	100	100	96	62	92	93	60
NGS GeneReader	42	82	83	40	70	100	100	89	61	100	100	57
Cobas	42	91	91	42	80	100	100	92	65	93	100	65
Therascreen	25	90	86	33	50	100	100	82	50	100	100	58

Sensitivity and specificity of each methodology, for the detection of the original EGFR sensitizing considering the gold standard the original tissue specimen is presented in Table S6.

## 4 Discussion

Biomarker testing using liquid biopsies is becoming the standard of care in many clinical laboratories as a noninvasive procedure with a short turnaround time. However, studies assessing the agreement between different platforms and, more importantly, exploring biological and technical factors responsible for discordant results are still limited. Here, we present the results of an observational trial specifically designed to evaluate the agreement between seven methods used for the detection of *EGFR* mutations in the cfDNA. Samples from 72 patients with stage IV NSCLC progressing to a first‐ or second‐generation TKI were prospectively collected in 23 Spanish hospitals under a strict protocol. The study was monitored by the Spanish Lung Cancer Group (GECP) to minimize the variability due to preanalytical conditions. Overall, the proportion of observed agreement among all the methods for the detection of *EGFR* mutations was good and particularly high for NGS‐based and high‐sensitivity PCR platforms. Interestingly, concordance was slightly lower for T790M mutation compared to mutations in exons 19 and 21, which always presented higher MAFs (*P* < 0.001; Wilcoxon test) (Fig. S4), consistent with the idea that T790M mutation is subclonal and arises later in tumor evolution and suggesting that the lower MAF values for T790M mutation could limit assay performance. A cross‐platform comparison including 38 samples from patients with *EGFR*‐mutated lung cancer from the phase 1 AURA trial analyzed the concordance between the results obtained with BEAMing, ddPCR, Therascreen, and cobas^®^ *EGFR* Mutation Test using tissue results as a nonreference standard [22]. Consistent with our results, the authors found that the concordance was lower for T790M (57%, 48%, 74%, and 70% for T790M vs 97%, 95%, 97%, and 95% for the L858R mutation using cobas, Therascreen, ddPCR, and BEAMing, respectively). In a recent study analyzing *EGFR* mutations in plasma from patients recruited in the AURA3 trial using the cobas *EGFR* Mutation Test v2 (cobas plasma), droplet digital polymerase chain reaction (ddPCR), and an NGS‐based test (Guardant360), a lower positive percent agreement was observed for the detection of T790M mutation compared with *EGFR* exon 19 deletion or L858R mutation [23].

In our study, rebiopsies were obtained from 35 (48.61%) patients, highlighting the limited tissue availability after disease progression due to patient safety concerns. Of note, the T790M mutation was detected in 18 cases in which rebiopsy was not feasible.

Considering tissue genotyping as a nonreference standard, in our study sensitivity ranged from 25% to 58% for the detection of T790M mutation. Consistent with our results, T790M mutation detection rates in blood samples collected upon disease progression have been reported to range as broadly as from 18% to 78% [10]. Specificity, however, was acceptable for real‐world applications, which supports the use of blood as a first choice for assessing *EGFR* mutations and relegating tissue tests to cases of negative plasma results. NGS, dPCR, PNA‐Q‐PCR, and BEAMing detected a higher number of T790M‐positive samples than cobas and Therascreen as was observed previously, but with fewer number or cross comparisons than in our study [10, 22, 23]. It is important to point out that EGFR‐sensitizing mutations landscape is complex and uncommon clinically relevant EGFR mutations in exons 18, 19, 20, 21 might not be detected by qPCR‐based technologies. In this way, singleplex approaches such as dPCR are limited by the number of mutations that can be interrogated in a given sample and might require to prioritize the order of mutations to be tested. On the contrary, NGS‐based approaches permit to interrogate a wide number of mutations simultaneously saving time and sample material.

We explicitly examined the effect of the input quantity DNA and MAF for discordant calls. First, we were not able to demonstrate an improvement in the agreement between methods when samples with low cfDNA input were discarded from the analysis. Conversely, the agreement ranged from almost perfect to perfect (*K* = 1) when excluding samples in which an *EGFR* mutation was detected at MAFs ≤ 0.5%. This observation could also explain the higher agreement and better sensitivity and specificity of methods when analyzing *EGFR*‐sensitizing mutation compared to the concomitant T790M mutation. As mentioned, the T790M mutation was always detected at lower MAFs with respect to the original *EGFR*‐sensitizing mutation. Consistent with this, in a recent paper comparing BEAMing and ddPCR for ctDNA analysis using plasma samples from advanced breast cancer patients enrolled in the PALOMA‐3 trial, the authors showed that discordant calls occurred at MAFs < 1% [24]. Likewise, in the above‐mentioned study analyzing samples from the AURA3 trial, the authors reported that discordant results between NGS and ddPCR always occurred in cases where mutations were detected at MAFs ≤ 1% [23]. Nevertheless, this study did not explore the patient's outcome according to MAFs, as it was conceived a non‐post‐authorization (non‐PAS), noninterventional study where participants did not receive treatment in relation to the study. Further studies addressing the impact on MAF in treatment outcome would be of particular interest.

We were not able to demonstrate a significant association between DNA input and discordant results, but the opposite hypothesis cannot be ruled out. Indeed, there was a significant but weak positive correlation between MAFs and total cfDNA concentration. Other researchers have previously reported this observation [25], meaning that *EGFR* mutations can be detected in the cfDNA at AF > 0.5%, even when the amount of cfDNA is low. Therefore, in our hands, MAFs is the best parameter for evaluating how trustworthy the result of a plasma test is and should be always informed in clinical reports. Clinical guidelines should stress the fact that plasma genotyping is less informative when MAFs are missing.

Despite emerging evidence suggesting that quantitative information from plasma genotyping is clinically relevant [24, 25], the available data evaluating the concordance between quantitative MAF values are still limited to small sets. Here, we compared MAF measurements assessed by each method using statistical methods common in laboratory medicine, namely the Passing‐Bablok regression and Bland–Altman plots. However, these methods are scarcely used for comparing the performance of molecular biology‐based techniques [26, 27]. Nevertheless, we believe that MAFs can be considered as a clinical chemistry analyte, such as glucose or cholesterol, and therefore, methods should be compared using statistical approaches specifically designed for this aim. Importantly, our data indicate that there was an excellent concordance between MAFs obtained by NGS‐based techniques and obtained by high‐sensitivity PCR‐based methods. With an analytic sensitivity like that of dPCR or BEAMing, NGS‐based assay would be the best option for studying oncogenic drivers in the ctDNA as a wider number of somatic mutations can be interrogated. A limited number of studies have evaluated the concordance between NGS‐based assays using clinical samples and conflicting results have been reported. While some researchers have found very low congruence between platforms for same‐patient‐paired samples [28], others have reported a high rate of concordance in direct comparisons between NGS‐based platforms, with discordant somatic mutations being mostly subclonal [29]. This suggests that discordant calls mostly occur at low MAF values.

Driver mutation MAFs varied from patient to patient by as much as 0.02% to 54.4%. To elucidate which factors could determine such a difference, we evaluated several clinical variables. In our hands, the presence of CNS metastasis was the only factor affecting MAFs, suggesting that biomarker testing using liquid biopsies could be limited by the anatomical location of the cancer lesions and that assay sensitivity might be compromised in patients with CNS metastasis. Indeed, it is well established that ctDNA is more frequently detected in patients with solid tumors outside the brain [30], and we have recently demonstrated that pleural effusion, ascites, and cerebrospinal fluid are superior to blood for detecting somatic mutations in patients with pleural, peritoneal, or CNS involvement [31]. On the other hand, since our cohort was very homogenous in terms of the type of patients included (i.e., all patients had stage IV disease, samples were all collected at the first disease progression after first‐line treatment with TKI, most cases were adenocarcinoma, etc.), we were unable to analyze the impact of other clinical factors affecting tumor shedding (i.e., tumor stage).

The strengths of our study include the comparison of many platforms, in contrast with previously published studies, sample anonymization and blind analysis by the participating laboratories and, finally, that samples were prospectively collected under a strict protocol, with all enrolled patients being at the same clinical treatment stage, immediately after progression to a first‐line TKI treatment. Moreover, the study was monitored by a contract research organization (Spanish Lung Cancer Group) to minimize clinical variability. One limitation is that we did not measure potential contamination from somatic mutations attributable to clonal hematopoiesis, although, to our knowledge, somatic mutations in the *EGFR* gene due to clonal hematopoiesis are very rare events at best [32, 33].

## 5 Conclusion

This prospective multicenter study demonstrates that NGS, digital PCR, and RT‐PCR‐based methodologies show good to excellent agreement for the detection of *EGFR* mutations in cfDNA, including the T790M mutation, with most discordant calls occurring at MAFs ≤ 0.5%. With NGS enabling the simultaneous testing of multiple mutations, our results support the use of this technology for noninvasive biomarker testing and suggest that MAFs and the limits of detection of the assay used should always be reported in the clinical setting.

## Conflict of interest

The authors have declared no conflict of interest at individual level. AstraZeneca supported this work. All reagents, necessary for the study, were funded by AstraZeneca. The study protocol and the final version of the manuscript were revised and approved by AstraZenca.

## Author contributions

AR, EJ‐L, MAM‐V conceived and coordinated the study. AR, EJ‐L, MAM‐V, MP, BG‐P, ARoy, SC‐F, AR‐F, and MP contributed to experimental design and data analysis. SC‐F, AR‐F, BG‐P, AI, PC, and AC carried out experiments. AR, ARoy, EJ‐L, and MAM‐V performed the statistical analyses. JPA, OJV, PD, MC, BH, SVE, GB, MG, MM, RB, ALO, AB, JB‐B, JMJ, JGG, SV, CGG, BM, and ALM selected the study population. AR, MAM‐V, and EJ‐L contributed to manuscript preparation. All authors read and approved the final manuscript.

## Ethics approval and consent to participate

The study was approved by the Hospital Puerta de Hierro Ethics Committee (internal code internal code PI‐154/17), and written informed consent was obtained from each participant.

## Supporting information


**Fig. S1.** Flowchart of the procedure work.
**Fig. S2.** Boxplot showing differences in MAFs between patients progressing at the brain level exclusively and patients with disease progression assessed at other anatomical locations.
**Fig. S3.** Comparison of MAFs obtained by NGS‐based platforms.
**Fig. S4.** Boxplot showing differences in AFs between sensitizing and the T790M mutation according to each platform.
**Table S1.** Input volume and elution volume according to extraction method.
**Table S2.** Analytical sensitivities for each platform.
**Table S3.** Total amount of cfDNA (ng) obtained per mL of plasma for each patient and according to each methodology.
**Table S4.** Agreement between methods for the detection of deletions in exon 19, point mutations in exon 21 and the T790M mutation.
**Table S5.** T790M detection rate in patients with and without CNS metastases according to method.
**Table S6.** Sensitivity, Specificity positive predictive value (PPV) and negative predictive value (NPV) of each methodology and according to the type of mutation and considering the gold standard tumor biopsy obtained at diagnosis.

**Data S1.** List of all patients included in the study showing mutation detected and corresponding mutant allele frequency according to methodology.

## Data Availability

Data are available upon request.

## References

[1] Sequist LV , Martins RG , Spigel D , Grunberg SM , Spira A , Jänne PA , Joshi VA , McCollum D , Evans TL , Muzikansky A *et al* (2008) First‐line gefitinib in patients with advanced non‐small‐cell lung cancer harboring somatic EGFR mutations. J Clin Oncol 26, 2442–2449. 18458038 10.1200/JCO.2007.14.8494

[2] Mok TS , Wu YL , Thongprasert S , Yang CH , Chu DT , Saijo N , Sunpaweravong P , Han B , Margono B , Ichinose Y *et al* (2009) Gefitinib or carboplatin‐paclitaxel in pulmonary adenocarcinoma. N Engl J Med 361, 947–957. 19692680 10.1056/NEJMoa0810699

[3] Rosell R , Carcereny E , Gervais R , Vergnenegre A , Massuti B , Felip E , Palmero R , Garcia‐Gomez R , Pallares C , Sanchez JM *et al* (2012) Erlotinib versus standard chemotherapy as first‐line treatment for European patients with advanced EGFR mutation‐positive non‐small‐cell lung cancer (EURTAC): a multicentre, open‐label, randomised phase 3 trial. Lancet Oncol 13, 239–246. 22285168 10.1016/S1470-2045(11)70393-X

[4] Park K , Tan E‐H , O'Byrne K , Zhang L , Boyer M , Mok T , Hirsh V , Yang JC‐H , Lee KH , Lu S *et al* (2016) Afatinib versus gefitinib as first‐line treatment of patients with EGFR mutation‐positive non‐small‐cell lung cancer (LUX‐Lung 7): a phase 2B, open‐label, randomised controlled trial. Lancet Oncol 17, 577–589. 27083334 10.1016/S1470-2045(16)30033-X

[5] Mok TS , Wu Y‐L , Ahn M‐J , Garassino MC , Kim HR , Ramalingam SS , Shepherd FA , He Y , Akamatsu H , Theelen WS *et al* (2017) Osimertinib or platinum‐pemetrexed in *EGFR* T790M–positive lung cancer. N Engl J Med 376, 629–640. 27959700 10.1056/NEJMoa1612674 PMC6762027

[6] Soria JC , Ohe Y , Vansteenkiste J , Reungwetwattana T , Chewaskulyong B , Lee KH , Dechaphunkul A , Imamura F , Nogami N , Kurata T *et al* (2018) Osimertinib in untreated EGFR‐mutated advanced non‐small‐cell lung cancer. N Engl J Med 378, 113–125. 29151359 10.1056/NEJMoa1713137

[7] Rolfo C , Mack PC , Scagliotti GV , Baas P , Barlesi F , Bivona TG , Herbst RS , Mok TS , Peled N , Pirker R *et al* (2018) Liquid biopsy for advanced non‐small cell lung cancer (NSCLC): a statement paper from the IASLC. J Thorac Oncol 13, 1248–1268. 29885479 10.1016/j.jtho.2018.05.030

[8] Leighl NB , Page RD , Raymond VM , Daniel DB , Divers SG , Reckamp KL , Villalona‐Calero MA , Dix D , Odegaard JI , Lanman RB *et al* (2019) Clinical utility of comprehensive cell‐free DNA analysis to identify genomic biomarkers in patients with newly diagnosed metastatic non‐small cell lung cancer. Clin Cancer Res 25, 4691–4700. 30988079 10.1158/1078-0432.CCR-19-0624

[9] Lung Cancer Metastatic. Available online at NCCN.org/patients NCCN guidelines for patients non‐small cell lung cancer.

[10] Mayo‐de‐las‐Casas C , Jordana‐Ariza N , Garzón‐Ibañez M , Balada‐Bel A , Bertrán‐Alamillo J , Viteri‐Ramírez S , Reguart N , Muñoz‐Quintana MA , Lianes‐Barragan P , Camps C *et al* (2017) Large scale, prospective screening of EGFR mutations in the blood of advanced NSCLC patients to guide treatment decisions. Ann Oncol 28, 2248–2255. 28911086 10.1093/annonc/mdx288

[11] Oxnard GR , Paweletz CP , Kuang Y , Mach SL , O'Connell A , Messineo MM , Luke JJ , Butaney M , Kirschmeier P , Jackman DM *et al* (2014) Noninvasive detection of response and resistance in EGFR mutant lung cancer using quantitative next‐generation genotyping of cell‐free plasma DNA. Clin Cancer Res 20, 1698–1705. 24429876 10.1158/1078-0432.CCR-13-2482 PMC3959249

[12] Jenkins S , Yang JCH , Ramalingam SS , Yu K , Patel S , Weston S , Hodge R , Cantarini M , Jänne PA , Mitsudomi T *et al* (2017) Plasma ctDNA analysis for detection of the EGFR T790M mutation in patients with advanced non‐small cell lung cancer. J Thorac Oncol 12, 1061–1070. 28428148 10.1016/j.jtho.2017.04.003

[13] Provencio M , Torrente M , Calvo V , Pérez‐Callejo D , Gutiérrez L , Franco F , Pérez‐Barrios C , Barquín M , Royuela A , García‐García F *et al* (2018) Prognostic value of quantitative ctDNA levels in non small cell lung cancer patients. Oncotarget 9, 488–494. 29416630 10.18632/oncotarget.22470 PMC5787483

[14] Karachaliou N , Mayo‐De Las Casas C , Queralt C , De Aguirre I , Melloni B , Cardenal F , Garcia‐Gomez R , Massuti B , Sánchez JM , Porta R *et al* (2015) Association of EGFR L858R mutation in circulating free DNA with survival in the EURTAC trial. JAMA Oncol 1, 149–157. 26181014 10.1001/jamaoncol.2014.257

[15] Friedman M (1937) The use of ranks to avoid the assumption of normality implicit in the analysis of variance. J Am Stat Assoc 32, 675–701.

[16] Landis JR & Koch GG (1977) The measurement of observer agreement for categorical data. Biometrics 33, 159. 843571

[17] Lin LI‐K (1989) A concordance correlation coefficient to evaluate reproducibility. Biometrics 45, 255. 2720055

[18] Steichen TJ & Cox NJ (2002) A note on the concordance correlation coefficient. Stata J Promot Commun Stat Stata 2, 183–189.

[19] McBride GB (2005) A proposal for strength‐of‐agreement criteria for Lin's concordance correlation coefficient. NIWA Client Report, HAM2005‐062.

[20] Passing H & Bablok W (1983) A new biometrical procedure for testing the equality of measurements from two different analytical methods. Application of linear regression procedures for method comparison studies in Clinical Chemistry. Part I. J Clin Chem Clin Biochem 21, 709–720. 6655447 10.1515/cclm.1983.21.11.709

[21] Martin Bland J & Altman DG (1986) Statistical methods for assessing agreement between two methods of clinical measurement. Lancet 327, 307–310. 2868172

[22] Thress KS , Brant R , Carr TH , Dearden S , Jenkins S , Brown H , Hammett T , Cantarini M and Barrett JC (2015) EGFR mutation detection in ctDNA from NSCLC patient plasma: a cross‐platform comparison of leading technologies to support the clinical development of AZD9291. Lung Cancer 90, 509–515. 26494259 10.1016/j.lungcan.2015.10.004

[23] Papadimitrakopoulou VA , Han JY , Ahn MJ , Ramalingam SS , Delmonte A , Hsia TC , Laskin J , Kim S‐W , He Y , Tsai C‐M *et al* (2020) Epidermal growth factor receptor mutation analysis in tissue and plasma from the AURA3 trial: osimertinib versus platinum‐pemetrexed for T790M mutation‐positive advanced non–small cell lung cancer. Cancer 126, 373–380. 31769875 10.1002/cncr.32503

[24] O'Leary B , Hrebien S , Beaney M , Fribbens C , Garcia‐Murillas I , Jiang J , Li Y , Huang Bartlett C , André F , Loibl S *et al* (2019) Comparison of beaming and droplet digital PCR for circulating tumor DNA analysis. Clin Chem 65, 1405–1413. 31551314 10.1373/clinchem.2019.305805

[25] Li BT , Janku F , Jung B , Hou C , Madwani K , Alden R , Razavi P , Reis‐Filho JS , Shen R , Isbell JM *et al* (2019) Ultra‐deep next‐generation sequencing of plasma cell‐free DNA in patients with advanced lung cancers: results from the actionable genome consortium. Ann Oncol 30, 597–603. 30891595 10.1093/annonc/mdz046 PMC6503621

[26] Provencio M , Pérez‐Barrios C , Barquin M , Calvo V , Franco F , Sánchez E , Sánchez R , Marsden D , Cristóbal Sánchez J , Martin Acosta P *et al* (2019) Next‐generation sequencing for tumor mutation quantification using liquid biopsies. Clin Chem Lab 58, 306–313. 10.1515/cclm-2019-0745 31469650

[27] Barquín M , Maximiano C , Pérez‐Barrios C , Sanchez‐Herrero E , Soriano M , Colmena M , García‐Espantaleón M , Tejerina González E , Gutierrez L , Sánchez Ruiz AC *et al* (2019) Peritoneal washing is an adequate source for somatic BRCA1/2 mutation testing in ovarian malignancies. Pathol Res Pract 215, 392–394. 30392916 10.1016/j.prp.2018.10.028

[28] Torga G & Pienta KJ (2018) Patient‐paired sample congruence between 2 commercial liquid biopsy tests. JAMA Oncol 4, 868–870. 29242909 10.1001/jamaoncol.2017.4027 PMC6145681

[29] Gupta R , Othman T , Chen C , Sandhu J , Ouyang C & Fakih M (2020) Guardant360 circulating tumor DNA assay is concordant with FoundationOne next‐generation sequencing in detecting actionable driver mutations in anti‐EGFR naive metastatic colorectal cancer. Oncologist 25, 235–243. 32162812 10.1634/theoncologist.2019-0441 PMC7066697

[30] Bettegowda C , Sausen M , Leary RJ , Kinde I , Wang Y , Agrawal N , Bartlett BR , Wang H , Luber B , Alani RM *et al* (2014) Detection of circulating tumor DNA in early‐ and late‐stage human malignancies. Sci Transl Med 6, 224ra24. 10.1126/scitranslmed.3007094 PMC4017867 24553385

[31] Villatoro S , Mayo‐de‐las‐Casas C , Jordana‐Ariza N , Viteri‐Ramírez S , Garzón‐Ibañez M , Moya‐Horno I , García‐Peláez B , González‐Cao M , Malapelle U , Balada‐Bel A *et al* (2019) Prospective detection of mutations in cerebrospinal fluid, pleural effusion, and ascites of advanced cancer patients to guide treatment decisions. Mol Oncol 13, 2633–2645. 31529604 10.1002/1878-0261.12574 PMC6887582

[32] Ptashkin RN , Mandelker DL , Coombs CC , Bolton K , Yelskaya Z , Hyman DM , Solit DB , Baselga J , Arcila ME , Ladanyi M *et al* (2018) Prevalence of clonal hematopoiesis mutations in tumor‐only clinical genomic profiling of solid tumors. JAMA Oncol 4, 1589–1593. 29872864 10.1001/jamaoncol.2018.2297 PMC6224316

[33] Coombs CC , Gillis NK , Tan X , Berg JS , Ball M , Balasis ME , Montgomery ND , Bolton KL , Parker JS , Mesa TE *et al* (2018) Identification of clonal hematopoiesis mutations in solid tumor patients undergoing unpaired next‐generation sequencing assays. Clin Cancer Res 24, 5918–5924. 29866652 10.1158/1078-0432.CCR-18-1201 PMC6812550

