# Supplementary material for: Comprehensive cross‐platform comparison of methods for non‐invasive EGFR mutation testing: results of the RING observational trial
Source: Mol Oncol. 2020 Nov 13;15(1):43–56. doi: 10.1002/1878-0261.12832 (PMC7782072; doi:10.1002/1878-0261.12832)
Supplement: Supplementary file 1 — Fig. S1. Flowchart of the procedure work. Fig. S2. Boxplot showing differences in MAFs between patients progressing at the brain level exclusively and patients with disease progression assessed at other anatomical locations. Fig. S3. Comparison of MAFs obtained by NGS‐based platforms. Fig. S4. Boxplot showing differences in AFs between sensitizing and the T790M mutation according to each platform. Table S1. Input volume and elution volume according to extraction method. Table S2. Analytical sensitivities for each platform. Table S3. Total amount of cfDNA (ng) obtained per mL of plasma for each patient and according to each methodology. Table S4. Agreement between methods for the detection of deletions in exon 19, point mutations in exon 21 and the T790M mutation. Table S5. T790M detection rate in patients with and without CNS metastases according to method. Table S6. Sensitivity, Specificity positive predictive value (PPV) and negative predictive value (NPV) of each methodology and according to the type of mutation and considering the gold standard tumor biopsy obtained at diagnosis. [file MOL2-15-43-s001.docx]

**Supplementary Information**

***Comprehensive cross-platform comparison of methods for non-invasive EGFR mutation testing, Results of the RING observational trial.***

*Atocha Romero,^1,2^ Eloisa Jantus-Lewintre,^3,4,5^ Beatriz García-Peláez,^6^ Ana Royuela,^7^ Amelia Insa,^8^*

*Patricia Cruz,^9^ Ana Collazo,^10^ Javier Pérez Altozano,^11^ Oscar Juan Vidal,^12^ Pilar Diz,^13^ Manuel*

*Cobo,^14^ Berta Hernández,^15^ Sergio Vázquez Estevez,^16^ Gretel Benítez,^17^ Maria Guirado,^18^*

*Margarita Majem,^19^ Reyes Bernabé,^20^* *Ana Laura Ortega,^21^ Ana Blasco,^3,^, Joaquim BoschBarrera,^22^ Jose M. Jurado,^23^ Jorge García González,^24^ Santiago Viteri,^25^ Carlos Garcia Giron,^26^*

*Bartomeu Massutí,^27^ Ana Lopez Martín,^28^ Alejandro Rodriguez-Festa,^1^ Silvia CalabuigFariñas,^3,4,29^ Miguel Ángel Molina-Vila,^6^ Mariano Provencio.^1,2^*

1. Liquid Biopsy Laboratory, Biomedical Sciences Research Institute Puerta de Hierro-Majadahonda
2. Medical Oncology Department, Hospital Universitario Puerta de Hierro-Majadahonda, Majadahonda ^3^ CIBERONC
3. Mixed Unit TRIAL, Príncipe Felipe Research Center & General University Hospital of Valencia Research Foundation, Valencia
4. Biotechnology Department, Universitat Politècnica de València, Valencia
5. Laboratory of Oncology/Pangaea Oncology, Quirón-Dexeus University Hospital, Barcelona
6. Biostatistics Unit, Hospital Universitario Puerta de Hierro-Majadahonda, Madrid, CIBERESP
7. Hospital Clínico Universitario de Valencia, Valencia
8. Hospital la Paz, Madrid
9. Hospital Universitario Sanchinarro, Madrid
10. Hospital Virgen de los Lirios, Valencia
11. Hospital Universitario La Fe, Valencia
12. Complejo Asistencial Universitario de León
13. Hospital Regional Universitario, Málaga
14. Complejo Hospitalario de Navarra, Navarra
15. Hospital Universitario Lucus Augusti, Lugo
16. Complejo Hospitalario Universitario Insular de Gran Canaria, Las Palmas
17. Hospital General Universitario de Elche, Elche, Alicante
18. Hospital de la Santa Creu i Sant Pau, Barcelona
19. Hospital Virgen del Rocío, Sevilla
20. Complejo Hospitalario de Jaén, Jaen
21. Hospital Dr. Josep Trueta-ICO Girona, Girona
22. Hospital Universitario Clínico San Cecilio, Granada
23. Hospital Clínico Universitario de Santiago, A Coruña
24. Instituto Oncológico Dr. Rosell, Hospital Universitario Dexeus, Grupo Quiron Salud, Barcelona
25. Hospital Universitario de Burgos
26. Hospital General Universitario Alicante
27. Hospital Severo Ochoa, Leganés, Madrid
28. Department of Pathology, Universitat de València, Valencia, Spain

# Corresponding authors, Atocha Romero (atocha10@hotmail.com), Eloisa Jantus-Lewintre (ejantus@btc.upv.es) and Miguel Ángel Molina (mamolina@panoncology.com) Supplementary Methods

|  | Input volume (mL) |  | Elution volume (ul) |
| --- | --- | --- | --- |
| Maxwell® RSC (MR) ccfDNA Plasma Kit ^1^ | 1.5 |  | 50 |
| QIAamp Circulating Nucleic Acid (Qiagen) | 4 |  | 140 |
| QIAsymphony DSP Virus/Pathogen Midi Kit | 4 |  | 50 |

^1^This extraction was performed twice

**Table S1.** Input volume and elution volume according to extraction method.

| ***Methodology*** |  |  | ***Mutation*** | |
| --- | --- | --- | --- | --- |
|  | ***Exon 19 deletion*** | ***L858R*** | ***T790M*** | ***Minimum DNA input (ng)*** |
| *Cobas® EGFR Mutation Test v2* | 1.4-13.4 | 5.3 | 3.0 | 50 |
| *Therascreen EGFR Plasma RGQ PCR Kit* | 0.81-10.4 | 5.9 | 17.5 | 7.5*^1^ |
| *QuantStudio® 3D Digital PCR System* | 0.1 | 0.1 | 0.1 | 20 |
| *PNA-Q-PCR* | 0.005 | 0.005 | 0.005 | 1 |
| *BEAMing (OncoBEAM Lung)* | 0.04 | 0.04 | 0.04 | NA*^2^ |
| *Oncomine Pan-Cancer Cell-Free Assay* | 0.1 | 0.1 | 0.1 | 20 |
| *QIAact Lung DNA UMI panel* | 0.4 | 0.4 | 0.4 | 10 |

^1^ The DNA input working range for the assay is defined by the control CT at the pre-specified range of 23.70 to 31.10.

*^2^For OncoBEAM Lung* DNA input in validation studies was ≥40 mutant molecules. Genome equivalents should range between 1000 and 10000 after amplification

**Table S2.** Analytical sensitivities for each platform. Limit of detection and input requirements as specified per manufacturer.

| ***Patient ID*** | ***QCNA*** | ***MR*** | ***QS*** |
| --- | --- | --- | --- |
| 100025 | 11.34 | 6.4 | 13.375 |
| 300046 | 17.78 | 4.2 | 9.25 |
| 300047 | 21.14 | 6.55 | 9.4 |
| 400003 | 10.99 | 9.5 | 11.375 |
| 400007 | 29.26 | 12.2 | 27.5 |
| 400033 | 60.13 | 23.75 | 40.5 |
| 400049 | 16.73 | 18.5 | 11.875 |
| 500038 | 23.1 | 4.65 | 19 |
| 500048 | 18.41 | 9.85 | 10.125 |
| 1000014 | 19.53 | 6 | 18.625 |
| 1000040 | 22.26 | 7.45 | 19.875 |
| 1000043 | 15.54 | 7.4 | 9.725 |
| 1300032 | 19.39 | 9.4 | 18.875 |
| 1300035 | 15.33 | 8.65 | 11.125 |
| 1300039 | 9.87 | 2.55 | 10.375 |
| 1300075 | 107.8 | 135 | 59.75 |
| 1700012 | 21 | 10.3 | 18 |
| 1700013 | 30.66 | 10.45 | 22.25 |
| 1700029 | 28.49 | 11.7 | 16.125 |
| 1700057 | 10.78 | 35.5 | 14.875 |
| 1700058 | 24.85 | 52.5 | 26.5 |
| 1700061 | 7.14 | 30.35 | 15.625 |
| 2100023 | 22.54 | 4.8 | 29.25 |
| 2100027 | 15.33 | 3.25 | 17.375 |
| 2300026 | 49.7 | 25 | 38.5 |
| 2300037 | 14.21 | 16.35 | 14.875 |
| 2300045 | 16.8 | 10.9 | 10.125 |
| 2800051 | 15.96 | 11 | 18.875 |
| 2800062 | 7.7 | 25.5 | 15.75 |
| 3500015 | 32.83 | 18.15 | 32.5 |
| 3500030 | 29.82 | 12.3 | 24.25 |
| 3500036 | 20.58 | 12.4 | 20.875 |
| 3500060 | 6.93 | 20.95 | 13.875 |
| 3500067 | 17.15 | 9.85 | 13.875 |
| 3600024 | 23.38 | 6.85 | 25.5 |
| 3600050 | 8.4 | 5 | 9.625 |
| 3600056 | 12.39 | 20.75 | 14 |
| 3600059 | 7.49 | 18.65 | 13.5 |
| 3600068 | 74.9 | 123 | 56.25 |
| 3700008 | 19.88 | 13.5 | 19.5 |
| 3700016 | 31.01 | 15.85 | 15.5 |
| 3700019 | 24.08 | 9.3 | 25.5 |
| 3700066 | 25.41 | 39.25 | 22.75 |
| 3700071 | 15.96 | 19.55 | 15.75 |
| 3900017 | 10.08 | 4.9 | 15.5 |
| 3900064 | 45.92 | 37.05 | 30.5 |
| 3900065 | 9.38 | 6.9 | 10.5 |
| 3900069 | 25.9 | 36.2 | 26.75 |
| 3900074 | 26.81 | 32.75 | 21.625 |
| 4700054 | 30.24 | 60.5 | 29.75 |
| 4700070 | 319.9 | 525 | 260 |
| 5300044 | 34.09 | 10.35 | 12.1 |
| 5300053 | 11.13 | 20.5 | 13.5 |
| 6200055 | 21.98 | 39.5 | 23 |
| 7200021 | 13.02 | 12.6 | 11.5 |
| 7200028 | 16.24 | 2.85 | 21.75 |
| 7200063 | 11.55 | 4.45 | 13.375 |
| 7200072 | 38.92 | 43.85 | 28.75 |
| 7800034 | 11.27 | 12.45 | 7.875 |
| 7800041 | 12.74 | 2.6 | 13.5 |
| 8500002 | 79.8 | 34.6 | 46.75 |
| 9400001 | 24.78 | 16.4 | 18.5 |
| 9400005 | 27.16 | 11.35 | 21.625 |
| 9400006 | 15.54 | 3.7 | 16.25 |
| 9400042 | 18.9 | 5.55 | 11.3 |
| 10100031 | 38.08 | 19.5 | 26.75 |
| 10100073 | 17.08 | 15.1 | 16.75 |
| 14600009 | 20.93 | 6.35 | 19 |
| 14600010 | 205.8 | 40.15 | 136.25 |
| 14600011 | 16.24 | 0.6 | 20.75 |
| 14600018 | 11.62 | 3.9 | 10.375 |
| 14600020 | 90.3 | 51.5 | 61.75 |

**Table S3. Total amount of cfDNA (**ng) obtained per mL of plasma for each patient and according to each methodology

| Comparision group | Mutation | Kappa | 95% CI | Kappa (excluding samples ≤ 0.5%) | 95% CI |
| --- | --- | --- | --- | --- | --- |
| High-sensitivity PCR based | exon 19 | 0.88 | 0.77-0.98 | 1 | 1.00-1.00 |
|  | exon 21 | 0.81 | 0.69-0.93 | 0.92 | 0.81-1.00 |
|  | T790M | 0.68 | 0.54-0.83 | 1 | 1.0-1.00 |
| NGS-based | exon 19 | 0.88 | 0.73-1.00 | 0.87 | 0.72-1.00 |
|  | exon 21 | 0.83 | 0.65-1.00 | 1 | 0.94-1.00 |
|  | T790M | 0.77 | 0.59-0.95 | 0.79 | 0.61-0.97 |

**Table S4.** Agreement between methods for the detection of deletions in exon 19, point mutations in exon 21 and the T790M mutation. Agreement excluding positive samples with AF ≤ 0.5%.

|  | T790M positive cases |  |  |
| --- | --- | --- | --- |
| ***Methodology*** | ***Progression at other locations*** | ***Progression at CNS*** | P |
| *BEAMing* | 41.67% | 16.67% | NS |
| *dPCR* | 45.00% | 8.33% | 0.046 |
| *PNA-Q-PCR* | 40.00% | 25.00% | NS |
| *NGS Oncomine* | 27.12% | 8.33% | NS |
| *NGS GeneReader* | 28.33% | 8.33% | NS |
| *Cobas* | 26.67% | 8.33% | NS |
| *Therascreen* | 22.41% | 0.00% | NS |

**Table S5.** T790M detection rate in patients with and without CNS metastases according to method.

|  | exon 21 |  |  |  | exon 19 |  |  |  |
| --- | --- | --- | --- | --- | --- | --- | --- | --- |
| ***Methodology*** | ***Sensitivity (%)*** | ***Specificity(%)*** | ***PPV (%)*** | ***NNV(%)*** | ***Sensitivity (%)*** | ***Specificity(%)*** | ***PPV (%)*** | ***NNV(%)*** |
| *Beaming* | 64 | 100 | 100 | 77 | 57 | 100 | 100 | 71 |
| *dPCR* | 70 | 100 | 100 | 79 | 50 | 100 | 100 | 69 |
| *PNA-Q-PCR* | 47 | 100 | 100 | 70 | 42 | 100 | 100 | 66 |
| *NGS Oncomine* | 53 | 100 | 100 | 72 | 49 | 97 | 94 | 66 |
| *NGS GeneReader* | 47 | 100 | 100 | 70 | 40 | 100 | 100 | 64 |
| *Cobas* | 70 | 100 | 100 | 80 | 57 | 97 | 95 | 71 |
| *Therascreen* | 37 | 100 | 100 | 66 | 44 | 100 | 100 | 66 |

**Table S6.** Sensitivity, Specificity positive predictive value (PPV) and negative predictive value (NPV) of each methodology and according to the type of mutation and considering the gold standard tumor biopsy obtained at diagnosis.


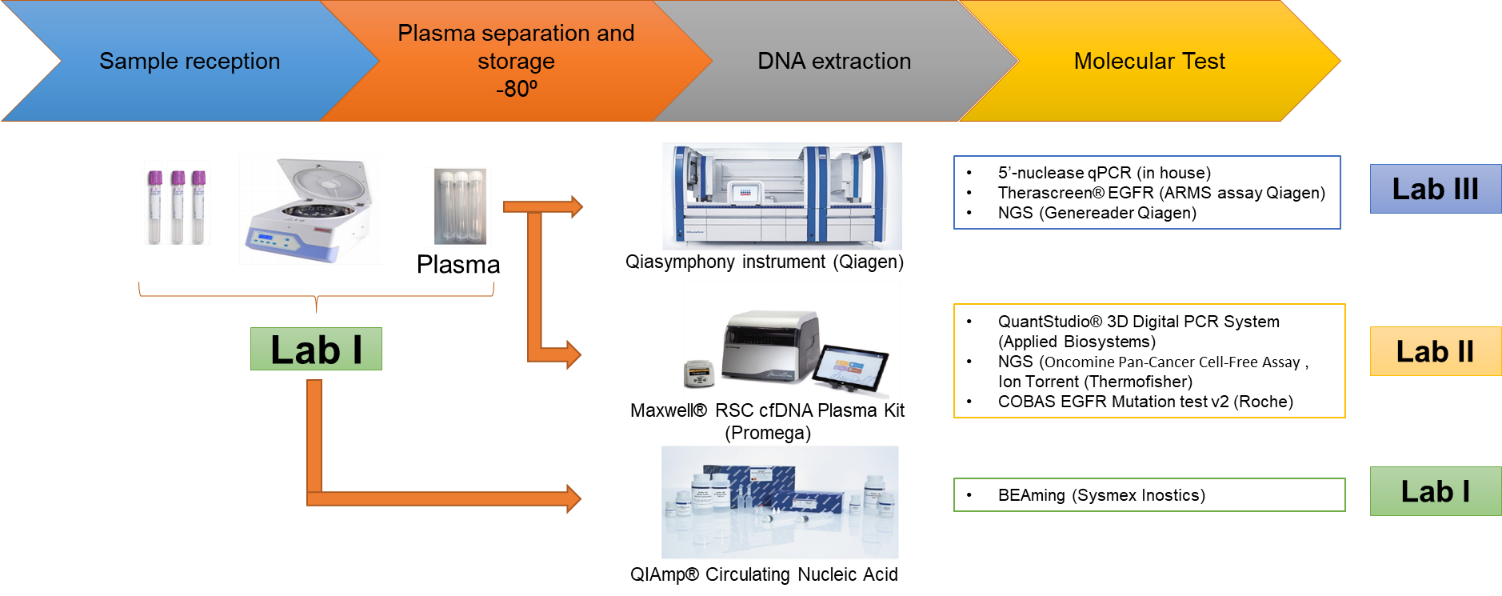


**Figure S1.** Flowchart of the procedure work. Lab I = Lab Oncología Molecular, Fundación Htal. Gral. Univ de Valencia Lab II = Lab de Biopsia Líquida, Fundación del Htal. Univ. Puerta de Hierro Lab III = Pangaea Oncology.


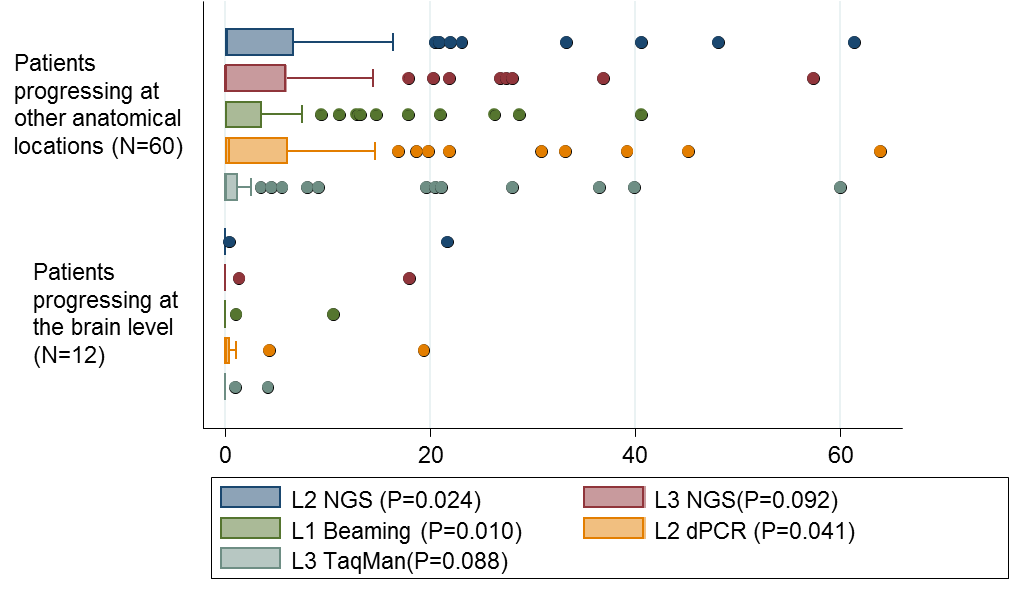


**Figure S2.** Boxplot showing differences in MAFs between patients progressing at the brain level exclusively and patients with disease progression assessed at other anatomical locations.


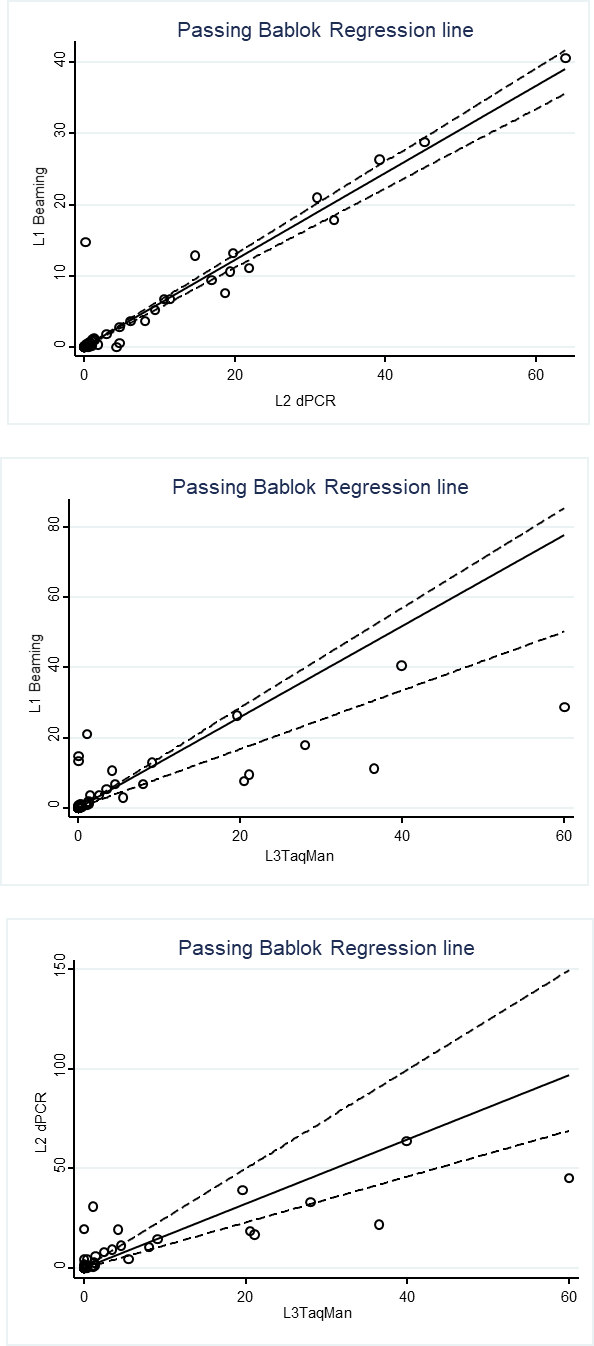


**Figure S3.** Comparison of MAFs obtained by NGS-based platforms. A. Passing-Bablok regression showing the concordance between methods for the assessment of EGFR-sensitizing MAFs. L1 = Lab Oncología Molecular, Fundación Htal. Gral. Univ de Valencia L2 = Lab de Biopsia Líquida, Fundación del Htal. Univ. Puerta de Hierro L3 = Pangaea Oncology.


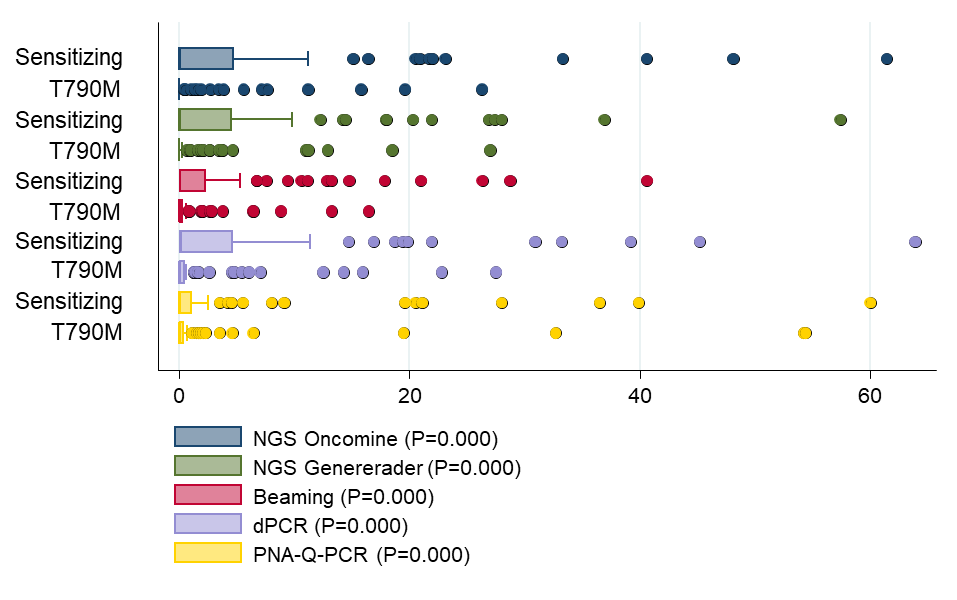


**Figure S4.** Boxplot showing differences in AFs between sensitizing and the T790M mutation according to each platform.
